# Facial Expression-based Pain Detection: Development and Validation in the Post-anesthesia Care Unit

**DOI:** 10.5812/aapm-171818

**Published:** 2026-04-30

**Authors:** Fazel Tarkhan, Ali Bijani, Shayan Alijanpour, Seyed Hossein Hamidi

**Affiliations:** 1 Clinical Research Development Unite of Rouhani Hospital, Babol University of Medical Sciences Babol Iran; 2 Social Determinants of Health (SDH) Research Centre, Health Research Institute, Babol University of Medical Sciences Babol Iran; 3 Department of Nursing, School of Nursing and Midwifery, Babol University of Medical Sciences Babol Iran; 4 Department of Anesthesiology, School of Medicine, Babol University of Medical Sciences Babol Iran

**Keywords:** Postoperative Pain, Facial Expression Analysis, Deep Learning, Pain Measurement, Automated Monitoring

## Abstract

**Background:**

Objective pain assessment in non-communicative patients remains challenging. Facial expressions are valid indicators of pain; however, manual coding is not practical.

**Objectives:**

This study aimed to develop and validate a real-time facial pain assessment system (FPAS) for detecting pain using deep learning-based facial expression analysis.

**Methods:**

This cross-sectional study enrolled 50 postoperative patients in the post-anesthesia care unit (PACU) of a teaching hospital. The system performed parallel processing of facial action units using FaceMesh and emotion recognition using DeepFace, with individual baseline calibration and temporal stabilization. At 1, 5, 10, 15, and 30 minutes after PACU admission, the system generated a 0 - 10 Visual Analog Scale (VAS) pain score and a four-level categorical classification, which were simultaneously compared with patients’ self-reported VAS ratings. Pearson correlation, the intraclass correlation coefficient (ICC), linear mixed-effects models, and classification metrics were used.

**Results:**

Fifty patients (60% female; mean age, 44.6 years) were enrolled. The Pearson correlation between the FPAS and patient self-reported VAS scores was 0.74 (P < 0.001). The ICC was 0.71 (95% CI: 0.64 - 0.77). Pain scores decreased significantly over time with both methods (P < 0.001), and the time × method interaction was not significant (P = 0.11). Categorical emotion classification showed 56% overall accuracy (Cohen’s kappa = 0.43, P < 0.001), with the greatest agreement for the neutral class (sensitivity 72%, specificity 85%). For the four-level pain intensity classification, overall accuracy was 78.5%, with sensitivity ranging from 44% (severe pain) to 85% (no pain).

**Conclusions:**

The automated FPAS demonstrated a strong correlation and acceptable agreement with patient self-reported VAS scores for postoperative pain monitoring in the PACU, supporting its potential as an adjunctive assessment tool. However, broader validation in larger, more diverse cohorts is needed before its clinical implementation.

## 1. Background

Pain is a multidimensional, subjective experience and remains one of the most prevalent and clinically challenging phenomena to quantify objectively (1). The International Association for the Study of Pain (IASP) defines pain as "an unpleasant sensory and emotional experience associated with, or resembling that associated with, actual or potential tissue damage" (1). Despite its universality, accurate pain assessment continues to pose substantial challenges in clinical practice, particularly for patients who cannot communicate their discomfort verbally, such as those with dementia, cognitive impairment, aphasia, or individuals under sedation (2).

Traditional pain assessment methods rely heavily on self-report instruments, including the Visual Analog Scale (VAS), Numeric Rating Scale (NRS), and the Wong-Baker Faces Pain Scale. While self-report remains the gold standard when feasible, it is inherently subjective, prone to recall bias, and influenced by cultural, psychological, and contextual factors (3). Patients may underreport or overreport pain because of a confluence of physical, psychological, motivational, and other factors, resulting in substantial variability in individual pain perceptions (4).

Nurses and physicians often disagree with parent or patient proxy reports of pain, and healthcare professionals may systematically underestimate the severity of patients' pain (5). McGaffin et al. reported that pain assessment in long-term care settings was characterized by inconsistent documentation, variability in the use of PRN analgesia, and organizational barriers to effective pain management, reinforcing concerns about the under-recognition and undertreatment of pain in institutional care (6). Behavioral scientists have long recognized that facial expressions provide reliable and valid indicators of pain (7). The Facial Action Coding System (FACS), developed by Ekman and Friesen, provides an objective, anatomically based coding system that categorizes facial expressions using a comprehensive operational description of specific actions. Research has identified a core set of facial Action Units (AUs) associated with pain expression, including brow lowering (AU4), cheek raising (AU6), lid tightening (AU7), nose wrinkling (AU9), upper lip raising (AU10), and lip corner pulling (AU12) (8). These facial movements occur spontaneously during painful experiences and are largely beyond conscious control, making them potentially more objective indicators than self-reported pain scores. Studies have demonstrated that facial expressions can discriminate painful from non-painful movements and that pain expression indices demonstrate test-retest reliability and concurrent validity with self-reports of pain (9). However, manual FACS coding requires extensive training, is time-consuming, and is impractical for real-time clinical use, creating a critical need for automated solutions (10).

Recent advances in computer vision, machine learning, and deep learning have catalyzed the development of automated facial expression analysis systems for pain assessment (11). Tools such as the Electronic Pain Assessment Tool (ePAT) and PainChek use automated facial recognition and algorithmic scoring to detect micro-expressions associated with pain, offering real-time, objective data to support clinical decision-making (12). These technologies claim to provide efficient, reproducible, and time-saving assessments that can be integrated into electronic health records, potentially reducing missed diagnoses and improving patient outcomes (6).

Pain assessment in nursing remains challenging because current methods are intermittent and subjective, and automated systems have important limitations, including small datasets, binary classification, and a lack of individual calibration. These limitations leave nurses without a reliable, non-invasive, continuous tool that works with standard bedside equipment and aligns with the biopsychosocial model of pain. The proposed system addresses this need by delivering real-time facial expression analysis from standard video cameras; hierarchically integrating Action Unit detection with deep learning-based emotion recognition; incorporating personalized baseline calibration and temporal stabilization; and providing verifiable data logging, including interface screenshots, thereby enabling a transition from laboratory prototypes to a clinically trustworthy tool for inpatient nursing environments.

## 2. Objectives

Therefore, this study aimed to develop and validate a novel facial pain assessment system (FPAS) that integrates multi-stream parallel processing (facial geometry/AU analysis and emotion recognition), individual baseline calibration, temporal stabilization, and standardized research-grade data logging, and to evaluate its performance against self-reported pain ratings and observer assessments in a clinical population.

## 3. Methods

### 3.1. Study Design and Setting

This cross-sectional study was conducted in the post-anesthesia care unit (PACU) of Ayatollah Rohani Hospital, affiliated with Babol University of Medical Sciences. The 30-day study period was from January 2026 to February 2026. The study protocol was approved by the institutional research ethics committee, and all necessary permissions were obtained before patient enrollment.

### 3.2. Study Population and Sample Size

The study population comprised all postoperative patients admitted to the PACU during a predefined period. Consecutive convenience sampling was used: Every eligible patient who entered the PACU during the study period was enrolled. The primary objective was to evaluate the correlation between pain intensity scores assigned by a physician using a VAS and those generated by a facial expression-based deep learning system. The sample size was calculated a priori using Pearson correlation testing. Assuming an expected correlation coefficient of 0.6 (indicating moderate-to-strong correlation), a significance level of 0.05, and a power of 80%, the minimum required sample size was estimated to be 45 patients. To account for potential dropout or incomplete data, an additional 10% was added, yielding a final sample of 50 patients. Of the 68 patients screened, 12 did not meet the inclusion criteria (10 had a Glasgow Coma Scale score < 15, and 2 declined consent), and 6 were excluded after enrollment (3 due to technical recording failures and 3 due to the administration of rescue opioids), resulting in a final analytic sample of 50 patients.

### 3.3. Inclusion and Exclusion Criteria

The inclusion criteria were: age 18 - 66 years; admission to the PACU after surgery with full consciousness; ability to cooperate with pain assessments; provision of written informed consent by the patient or legal guardian; no history of facial muscle dysfunction (e.g., facial paralysis); no severe psychiatric disorder that would preclude understanding simple commands; no history of interventions affecting facial expression (e.g., botox); and a Glasgow Coma Scale score of 15 at the time of assessment (indicating full alertness and the ability to produce facial expressions). The exclusion criteria were: withdrawal of consent by the patient or legal guardian at any stage; inability to record or correctly analyze facial images due to technical issues or excessive motion; development of a new medical or psychiatric condition impairing cooperation; incomplete data collection; identification of previously unreported facial dysfunction (e.g., facial paralysis) after enrollment; facial surgery; and administration of opioid analgesics after PACU admission. Regarding perioperative analgesic exposure, patients were not excluded based on preoperative or intraoperative analgesics, as these were standardized per protocol. For patients undergoing general anesthesia exceeding 90 minutes in duration, the anesthesia team was permitted to administer a single additional intraoperative fentanyl bolus of 0.5 - 1 μg/kg IV if clinically indicated; these patients were not excluded, as this reflects standard clinical practice. Any patient requiring rescue opioid analgesics during the 30-minute PACU observation period was excluded from the analysis to avoid confounding the assessment of the system’s ability to detect pain without opioid-induced blunting of facial expression.

### 3.4. Data Collection and Study Procedure

Before formal data recording, a brief baseline stabilization phase (the first few seconds) was implemented for system calibration and to capture each patient’s individual resting facial pattern. This baseline was used only to improve facial analysis accuracy and was not used in pain score calculations at the primary time points. Real-time assessments were performed simultaneously by both methods at 1, 5, 10, 15, and 30 minutes after PACU admission. The timing of these assessments relative to analgesic administration was as follows: intraoperative acetaminophen was administered approximately 30 - 60 minutes before PACU arrival; intraoperative fentanyl (when used) was administered at induction, typically 60 - 120 minutes before the first assessment. For patients requiring an additional fentanyl bolus during prolonged surgery, the assessment timing was calculated from the time of the last fentanyl administration, ensuring that all assessments occurred at least 60 minutes after the final opioid dose. No analgesics were administered during the 30-minute observation period. Therefore, all pain assessments occurred during the washout/declining phase of intraoperative analgesics, without confounding by new analgesic administration during the study window.

The physician first asked the patient to self-report the current pain level using a standard 0 - 10 VAS. For this conscious and cooperative cohort, the patient’s self-reported VAS was recorded as the reference standard (patient self-reported VAS rating). The physician was blinded to the FPAS output throughout the data collection period. The FPAS provided 1) a continuous 0 - 10 pain intensity score; 2) a primary four-level categorical pain intensity classification (No Pain [VAS 0], Mild [VAS 1 - 3], Moderate [VAS 4 - 6], Severe [VAS 7 - 10]); and 3) a secondary, exploratory seven-class emotion recognition output (neutral, happy, sad, angry, fear, disgust, surprise). The emotion recognition output was not part of the primary validation and is reported separately.

### 3.5. Perioperative Analgesic Protocol

All patients followed a standardized institutional analgesia protocol. Preoperatively, no patient received sedative or analgesic premedication. Intraoperatively, all patients received intravenous acetaminophen (1 g) administered 30 minutes before the end of surgery. Additionally, patients undergoing general anesthesia (n = 40; 80%) received intraoperative fentanyl 1 - 2 μg/kg IV at induction, with no additional scheduled intraoperative opioids; however, for surgeries lasting > 90 minutes, a single supplemental fentanyl bolus (0.5 - 1 μg/kg IV) was permitted as clinically indicated. Patients receiving spinal/epidural anesthesia (n = 10; 20%) received intrathecal bupivacaine 12 - 15 mg without intrathecal opioids. No intraoperative non-steroidal anti-inflammatory drugs (NSAIDs) or ketamine were administered. In the PACU, no analgesic medications (including opioids, NSAIDs, or rescue acetaminophen) were administered during the 30-minute observation period per protocol. Patients who required rescue analgesia (defined as any opioid, e.g., morphine 2 - 4 mg IV, for VAS > 7) during the observation period were excluded from the final analysis, as specified in the exclusion criteria. Thus, the reported pain scores reflect the natural postoperative pain trajectory without pharmacological intervention during the assessment window.

### 3.6. System Overview

We developed a real-time, video-based pain assessment system that operates online using a patient’s facial video stream. The system performs multilayer parallel analyses on the live video feed, outputs a pain score on the VAS, and simultaneously generates a standardized data acquisition protocol for research purposes. The architecture is designed to generate both a user-observable output (real-time VAS) and a citable, traceable research output, in which every major system decision is recorded and can be reviewed. The system does not merely display a number; it also logs all relevant events, including baseline registrations, scheduled captures, manual operator requests, and associated image files, enabling full reproducibility.

### 3.7. Patient-Specific Data Organization

Data were organized by unique patient identifiers into dedicated folders, with a CSV event log documenting timestamps and event types. High-pain clinical alerts were logged independently for rapid review.

### 3.8. System Parameters

The system applies three main processing streams to the same camera image, all executed in real time (Figure 1).

**Figure 1.**
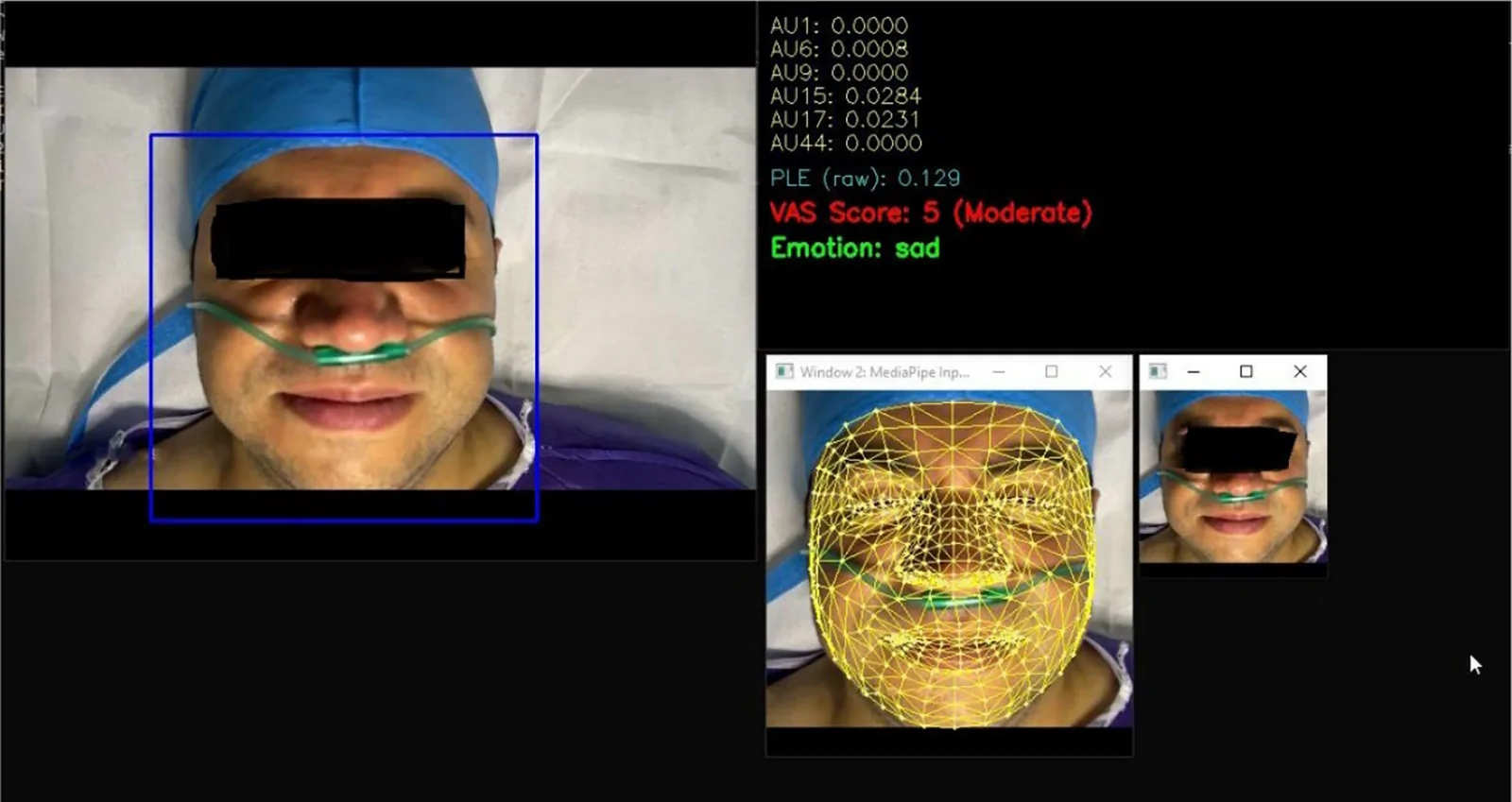
Facial expression analysis for pain detection.

#### 3.8.1. Stream 1: Face Detection and ROI Stabilization

The first stream detects the face and defines the region of interest (ROI). Rather than analyzing the entire frame, the system locates the face and restricts subsequent processing to the facial ROI. This approach increases processing speed (as only a smaller region is analyzed) and improves quality by focusing subsequent models on the face. Importantly, the ROI is stabilized using temporal smoothing to prevent jitter: if the detector slightly shifts the bounding box between frames, the ROI does not oscillate, as such oscillations would directly introduce noise into the outputs of later layers.

#### 3.8.2. Stream 2: Facial Geometry and Muscle Cues via FaceMesh

The second stream extracts facial geometry and muscle cues using FaceMesh, which provides a dense set of facial landmarks. The system computes meaningful distances and ratios from these landmarks within specific facial regions (e.g., around the eyes, eyebrows, nose, mouth, and jaw). These calculations serve as direct physiological/biomechanical features that closely approximate facial muscle contractions. The system models these features in terms of AUs. The objective is not to label the patient as “distressed,” but to identify specific patterns of muscle tension in the periocular, brow, nasal, perioral, and jaw regions—areas consistently associated with pain expression in the literature. The detected AUs are then weighted such that AUs known to be more indicative of pain contribute more strongly to the final pain score.

#### 3.8.3. Stream 3: Emotion Recognition via DeepFace

The third stream performs emotion recognition using DeepFace. Unlike the geometric features, this stream outputs an emotional label (e.g., neutral, sad, angry, fear, etc.). In this design, emotion does not replace AUs but serves as a modulatory layer. Specifically, the system first computes an AU-based pain intensity and then slightly amplifies or attenuates this intensity based on the detected emotion. The underlying rationale is that certain emotional states overlap with pain patterns or can exacerbate pain display, whereas other states (e.g., happiness) are generally less compatible with pain expression. Because raw emotion outputs can fluctuate from frame to frame, the system applies temporal smoothing and stabilization: it does not accept the emotion of the current frame alone but instead uses a short buffer to perform majority voting. A multi-frame confirmation mechanism is also implemented for emotion changes, preventing momentary label flips that would destabilize the pain output.

### 3.9. Individual Calibration (Baseline Stabilization Phase)

At system startup, an individual calibration phase is performed to avoid uniform behavior across subjects and to account for natural differences in facial morphology, camera distance, facial shape, and baseline expressions. During this phase, which lasts for a predefined number of frames, the system enters a stabilization state. No final VAS pain score is displayed to the user. Instead, the system collects facial data to determine what constitutes the “normal state of this specific individual under these specific imaging conditions.” Once the calibration phase is complete, a personalized baseline is constructed. From that moment onward, any sign of pain detected by the system is defined as a deviation from this individual baseline rather than as a raw absolute value. In engineering terms, this creates inter-subject normalization, reducing sensitivity to static individual differences and focusing on meaningful changes.

### 3.10. Operational Loop and Dynamic Pain Score Computation

After calibration, the operational loop processes each frame through five sequential stages: 1) face detection and AU intensity extraction using FaceMesh landmarks; 2) temporal smoothing of AUs via a 15-frame moving average to reduce noise; 3) construction of a raw pain score from weighted AUs, followed by dynamic scaling that tracks running min/max values with a decay factor of 0.95 to normalize scores to a 0 - 1 range; 4) emotion modulation using DeepFace-derived coefficients (ranging from 0.7 for happy to 1.3 for angry) and application of a low-pain threshold of 0.2 to eliminate false positives; and 5) mapping to a 0 - 10 VAS scale with exponential smoothing for stable output.

### 3.11. Slow Baseline Adaptation

A slow baseline adaptation mechanism is included and is activated only when the system detects sustained low pain levels. The purpose is to allow the baseline to adjust gradually if conditions change slightly during an assessment. Critically, when pain is high, the baseline does not change, preventing the system from gradually normalizing pain and losing sensitivity.

### 3.12. Visual Multi-Window Interface

The system produces a multi-window visual interface that displays real-time information and provides visual documentation for publications. The raw frame window shows the original video with the facial ROI marked, allowing immediate verification that the system has correctly located the face. The FaceMesh overlay window displays the dense landmark network superimposed on the face, which is essential for quality control; if landmarks slip or are incorrectly placed, this can be detected instantly. The DeepFace ROI window shows the cropped face region used for emotion recognition, clarifying which image is provided to the emotion classifier. The text information panel displays not only the VAS score and a qualitative pain description but also the raw AU values and the normalized pain score, improving explainability for research purposes rather than functioning as a black box.

### 3.13. Data Logging for Research

The system supports both scheduled (time-based) and manual (operator-initiated) data logging. Scheduled logging operates as a milestone mechanism: at specified time points (e.g., every N minutes), a set of screenshots is captured from all windows. This ensures a standardized, comparable dataset. For example, if a research protocol dictates documentation at minutes 5, 10, and 15, the system performs this automatically. Baseline logging is performed as a special event: immediately after calibration is complete and all displays are ready, a baseline capture is taken so that the reference point is preserved in the data. Manual logging is triggered when the operator presses a designated key at any time; the system then saves the current state of all windows and records the event in the CSV log. Crucially, the screenshot mechanism captures the actual desktop windows as displayed on the screen, rather than the image arrays in program memory. This means that, for publication purposes, one can show exactly what the operator saw (i.e., the actual user interface), providing greater verifiability.

### 3.14. Rigor

The code incorporates several conservative behaviors to prevent a single bad frame or model error from crashing the system. If DeepFace returns an error or an unexpected output, the system defaults to neutral and continues uninterrupted. If landmarks for certain points are unusable, the AU calculation is designed to ignore that portion and produce a safe default value rather than crashing. If insufficient data are available to determine a stable emotion, the system defaults to neutral to avoid premature decisions. High-pain events are logged separately, ensuring that even if the CSV is later used for statistical analysis, the clinical alert data are retained.

To ensure reproducibility, the following parameters are disclosed:

AU weights (Stream 2): AU4 (Brow Lowering) = 0.25, AU6 (Cheek Raising) = 0.20, AU7 (Lid Tightening) = 0.25, AU9 (Nose Wrinkling) = 0.15, AU10 (Upper Lip Raise) = 0.10, AU12 (Lip Corner Pull) = 0.05. Emotion modulation coefficients (Stream 3): Sad = 1.2, Angry = 1.3, Fear = 1.15, Disgust = 1.1, Neutral = 1.0, Happy = 0.7, Surprise = 0.9. Calibration: Baseline duration = 150 frames (~5 seconds at 30 fps). Temporal smoothing = moving average over 15 frames. Emotion change confirmation = majority vote over 10-frame buffer. Dynamic scaling: Running min/max tracking with a decay factor of 0.95. Low-pain threshold: Normalized pain scores below 0.2 (on a 0 - 1 scale) are floored to zero VAS. Hardware: Logitech C920 HD Pro webcam, 1920x1080 resolution, 30 fps, fixed distance of 0.75 - 1.0 meters from patient's face, consistent overhead ambient lighting (150 - 200 lux). Code availability: Pseudocode is available from the corresponding author upon reasonable request.

### 3.15. Outcome Measures and Validation

The reference standard was the patient’s self-reported VAS score obtained by a blinded physician. The FPAS generated continuous and categorical outputs. System accuracy was evaluated at two levels. First, for the continuous VAS-like output, correlation with patient self-reported VAS scores was assessed using Pearson or Spearman correlation and the intraclass correlation coefficient (ICC). Second, for the four-level pain intensity classification, the system’s classifications were compared with the patient’s VAS-derived category using accuracy, sensitivity, specificity, and F1 score. This dual approach was intended to provide a comprehensive assessment of system performance and to address the limitations of the traditional VAS.

### 3.16. Statistical Analysis

A linear mixed-effects model with patient as a random effect was used to account for the non-independence of repeated measurements (5 time points) nested within individual patients. Descriptive statistics, Pearson correlation, paired t-tests, repeated-measures ANOVA, ICC, chi-square tests, and classification metrics were used. Confidence intervals (95%) were calculated for all performance metrics. A significance level of P < 0.05 was considered statistically significant.

## 4. Results

A total of 50 patients (60% female) with a mean age of 44.6 years (SD = 13.2) were enrolled. No significant correlation was observed between pain scores and patient age (r = –0.18, P = 0.12). Mean pain scores did not differ significantly by sex (4.45 in females vs. 4.38 in males, P = 0.53). Surgical types included abdominal (n = 22; 44%), orthopedic (n = 18; 36%), and other (n = 10; 20%). General anesthesia was used in 40 patients (80%), and spinal/epidural anesthesia was used in 10 (20%). Per protocol, no patient received opioid analgesics during the 30-minute observation period.

Pain scores were recorded at five time points (1, 5, 10, 15, and 30 minutes after PACU admission) simultaneously by patient self-report and by the FPAS. Both methods showed a progressive decrease in pain intensity over time. The highest mean scores occurred at 1 minute (both methods: 6.12), and the lowest at 30 minutes (patient self-report: 2.82, machine: 2.72). The largest, and only statistically significant, difference was observed at 15 minutes, when the FPAS assigned higher pain scores than the self-report VAS (mean difference –0.46, P = 0.01). ANOVA showed a significant decrease in pain scores over time for both methods (P < 0.001). The time × method interaction was not significant (P = 0.11) (Table 1).

**Table 1.** Pain Scores by Assessment Method and Time Point ^a^

Time (min)	Patient VAS	FPAS	Mean Difference (Patient – System)	P-Value ^b^
**1**	6.12 ± 1.48	6.12 ± 1.65	0.00	0.99
**5**	5.18 ± 1.21	5.10 ± 1.44	0.08	0.68
**10**	4.22 ± 0.92	4.46 ± 1.31	-0.24	0.16
**15**	3.64 ± 1.05	4.10 ± 1.27	-0.46	0.01
**30**	2.82 ± 1.18	2.72 ± 1.39	0.10	0.57
**All times**	4.40 ± 1.52	4.50 ± 1.64	-0.10	0.33

^a^ Values are expressed as mean ± SD. Abbreviations: VAS, Visual Analog Scale; SD, standard deviation; FPAS, Facial Pain Assessment System.

^b^ P values were derived from paired t tests comparing patient self-reported VAS and FPAS scores at each time point. The combined P value represents the repeated-measures ANOVA comparison across all measurement points. Statistical significance was set at P < 0.05.

Across all 250 measurements, the Pearson correlation between the self-report VAS and the FPAS was r = 0.74 (P < 0.001), indicating a strong positive correlation. The ICC (two-way absolute agreement) was 0.71 (95% CI: 0.64 - 0.77), indicating acceptable reliability between the two assessment methods. The linear mixed-effects model, accounting for repeated measures within patients, confirmed a strong correlation (r = 0.74, P < 0.001).

For the primary four-level pain intensity classification, the system achieved an overall accuracy of 78.5%. Sensitivity was 85% for no pain, 72% for mild pain, 68% for moderate pain, and 44% for severe pain. Specificity ranged from 82% (mild pain) to 96% (severe pain). For the secondary seven-class emotion recognition, overall accuracy was 56% (Cohen's kappa = 0.43, P < 0.001), with the highest agreement for the neutral class (sensitivity 72%, specificity 85%) and the lowest for the happy class (n = 2; sensitivity, 0%) (Tables 2 and 3).

**Table 2.** Classification Performance of the Facial Pain Assessment System for Seven-Class Emotion Recognition ^a^

Emotion class	Sensitivity	Specificity	F1-score
**Neutral**	72	85	0.74
**Sad**	58	71	0.62
**Angry**	44	83	0.52
**Fear**	48	79	0.55
**Disgust**	35	92	0.46
**Surprise**	40	88	0.50
**Happy**	0	99	0.00

^a^ Values are expressed as percentage. Sensitivity = proportion of the actual emotion class correctly identified; specificity = proportion of nonclass emotions correctly excluded; F1 score = harmonic mean of precision and recall (range, 0 - 1, where 1 indicates perfect performance). Class imbalance was noted for the happy class (n = 2); this class should be interpreted cautiously.

**Table 3.** System Performance for Four-Level Pain Intensity Classification ^a^

Pain Category (VAS)	Accuracy	Sensitivity	Specificity	F1-Score
**No Pain (0)**	88	85	90	0.87
**Mild (1-3)**	71	68	73	0.70
**Moderate (4-6)**	69	72	66	0.69
**Severe (7-10)**	91	44	98	0.61
**Overall**	78.5	–	–	–

^a^ Values are expressed as percentage. Abbreviation: VAS = Visual Analog Scale. No pain = VAS 0; mild = VAS 1 - 3; moderate = VAS 4 - 6; severe = VAS 7 - 10. Sensitivity = proportion of the actual pain level correctly identified by the FPAS; specificity = proportion of nontarget pain levels correctly excluded; F1 score = harmonic mean of precision and recall (range, 0 - 1, where 1 indicates perfect classification). Overall accuracy = total correct classifications divided by total classifications across all pain levels.

## 5. Discussion

In this study, we developed and validated a real-time FPAS that integrates parallel processing of facial geometry (Action Units) and emotion recognition, individual baseline calibration, temporal stabilization, and standardized data logging. The system demonstrated a strong correlation (r = 0.74) and acceptable agreement (ICC = 0.71) with patient self-reported VAS scores. It achieved moderate performance in categorical emotion classification (overall accuracy 56%, Cohen’s kappa = 0.43), with pain-related emotion classes (sad, angry, fear) showing reasonable sensitivity. Both methods exhibited a significant decrease in pain intensity over time (P < 0.001), with no significant time × method interaction (P = 0.11), indicating that the system captured the temporal pain trajectory similarly to patient self-report.

The performance of our system (r = 0.74, ICC = 0.71) is comparable to that of previously reported automated pain assessment tools. Aydın and Özyazıcıoğlu, using OpenFace for facial action unit analysis in pediatric postoperative patients, reported that machine-predicted pain scores were closest to child self-reported pain among all raters (child, mother, nurse, observer) (9).

Their study demonstrated the feasibility of automated facial expression analysis for pain assessment, although specific correlation coefficients were not reported. In a prospective study of critically ill patients, Wu et al. developed a deep learning-based pain classifier using convolutional neural network (CNN) models and reported dichotomous classification accuracies of 0.7668 for pain vs. no-pain discrimination and 0.8593 for pain vs. severe pain classification (13). However, their study focused on binary classification rather than continuous pain intensity estimation across the full pain spectrum. The discrepancy in performance metrics between our study and Wu et al. likely arises from differences in outcome definitions: We evaluated correlation with continuous VAS-like outputs, whereas Wu et al. assessed categorical classification accuracy (13). Furthermore, our study enrolled postoperative patients in the PACU, a setting in which pain levels are generally lower and more variable than in the intensive care unit (ICU), potentially contributing to differences in observed performance. The EDi Pain model, a deep learning-based instance-appraisable model for pain estimation from facial videos, reported a Pearson correlation coefficient of 0.63 on the UNBC-McMaster dataset and 0.22 on a prospective emergency department dataset of 931 patients (10).

Our correlation coefficient of 0.74 exceeds the EDi Pain model’s performance on the UNBC dataset and substantially exceeds its prospective validation performance. This difference may be explained by several factors. First, our system incorporates individual baseline calibration, which accounts for inter-individual differences in facial morphology and baseline expression patterns, a feature absent in the EDi Pain model. Second, our emotion modulation layer, which applies emotion-specific weighting to pain intensity estimates, may have improved sensitivity to pain-related expressions by reducing false positives from non-pain facial movements. Third, our temporal smoothing and stabilization mechanisms likely reduced frame-to-frame noise, enhancing signal stability. Finally, the lower correlation in the EDi Pain prospective validation (r = 0.22) suggests that performance degrades substantially when moving from controlled to real-world clinical settings, highlighting the importance of validation in representative clinical populations, such as our PACU cohort. The Dual-Attention CNN (DA-CNN) framework achieved a test accuracy of 90.19% ± 0.94% on clinically annotated datasets, with an F1 score of 0.90 and Cohen’s κ = 0.876, demonstrating robust discrimination across five pain intensity levels (8).

This performance substantially exceeds our system’s categorical classification metrics (overall accuracy 56%, κ = 0.43). Several factors may account for this discrepancy. The DA-CNN model was trained and validated on a clinically annotated dataset in which ground-truth labels were likely derived from expert-coded PSPI scores or structured clinical assessments, providing a more objective and reliable reference standard than our physician VAS ratings, which are inherently subjective. Additionally, the DA-CNN framework employed multi-head spatial attention and triple-pooling channel attention to localize pain-relevant facial regions, representing a more sophisticated architecture than our weighted AU approach. The DA-CNN was also trained on larger, more standardized datasets (such as the UNBC-McMaster Shoulder Pain Expression Archive Database), whereas our system was developed and validated on a relatively smaller clinical dataset. The higher performance reported for DA-CNN reflects laboratory-controlled conditions, whereas our system was evaluated in a live clinical PACU setting with real-time video capture, where lighting, camera angles, and patient movement are less controlled. These differences highlight the well-recognized challenge of translating high-performing laboratory models to real-world clinical environments (14).

The weighted facial expression analysis model proposed by the 2025 study achieved an average accuracy of 92.72% using a weighted pain level estimation model, compared with 83.37% with conventional approaches, on a dataset of 4084 facial images from 25 individuals (11). This high accuracy, similar to that reported for the DA-CNN framework, reflects controlled experimental conditions. Our system’s moderate categorical classification accuracy (56%) and Cohen’s kappa = 0.43 are more consistent with real-world clinical validation studies. A systematic review of AI-based pain assessment via facial expression recognition (2015 - 2025) found that accuracy in pain detection varied between approximately 70% and > 90%, with higher performance observed in deep learning and multimodal frameworks, but noted that evidence remains limited by small sample sizes, methodological heterogeneity, and scarce external validation (14). Our findings align with this systematic review’s observation that performance degrades when moving from controlled laboratory settings to live clinical environments.

In terms of temporal analysis, repeated-measures ANOVA in our study revealed a significant decrease in pain scores over time for both methods (P < 0.001), with no significant time × method interaction (P = 0.11). This suggests that the system captured the expected postoperative pain trajectory (highest immediately after surgery with gradual decline) similarly to physician assessment. The absence of a significant interaction supports the clinical validity of the system’s dynamic pain monitoring capability. This finding is clinically important because it demonstrates that the system can detect not only static pain levels but also meaningful changes in pain intensity over time, a requirement for effective postoperative pain management.

The system’s categorical emotion classification performance (overall accuracy 56%, κ = 0.43, moderate agreement) warrants discussion. The highest agreement was observed for the "neutral" class (sensitivity 72%, specificity 85%), whereas the "happy" class showed no correct classifications (sensitivity 0%). Pain-related emotion classes (sad, angry, fear) achieved moderate sensitivity (44 - 58%). The poor performance for "happy" likely reflects class imbalance—only two observations of happy expressions in the dataset—rather than a genuine system limitation. The moderate sensitivity for pain-related emotions suggests that, while the system can detect some aspects of pain expression, there remains room for improvement in distinguishing pain from similar negative emotions. This is consistent with the literature, which notes that pain expressions can overlap with other negative affective states, making discrimination challenging (11).

Clinically, the relatively low sensitivity for severe pain detection (44%) is a notable limitation that warrants explicit discussion. Failure to identify severe pain cases may limit the current clinical applicability of the system, particularly in settings where prompt identification of high-intensity pain is critical for patient safety and timely intervention. This performance gap suggests that the system, in its current form, should be considered a supplementary screening tool rather than a replacement for clinical judgment. Nurses and physicians should not rely solely on the FPAS to detect severe pain but should use it as an adjunct to comprehensive clinical assessment.

Several limitations of this study should be acknowledged. First, the relatively small sample size limited our ability to perform stratified analyses and subgroup comparisons and precluded external validation on an independent dataset, thereby restricting generalizability. The reference standard—physician VAS ratings—was inherently subjective, and although a single rater ensured consistency, future studies should incorporate multiple raters with an inter-rater reliability assessment. The study was conducted exclusively in the PACU of a single hospital, potentially limiting applicability to other settings such as ICUs, emergency departments, or long-term care facilities. The system’s emotion classification performance was only moderate, and the weighted Action Unit approach, although literature grounded, may not fully capture the complexity and cultural variability of spontaneous facial pain expressions. The dataset contained relatively few severe pain events (VAS > 7), limiting assessment of system sensitivity to high-intensity pain. Technical factors, including camera resolution, frame rate, lighting, and patient positioning, were not systematically controlled or reported, which may have influenced performance. Finally, the data logging protocol, although standardized for research, may require adaptation for routine clinical use, where workflow integration and real-time alerting are paramount.

### 5.1. Conclusions

This study demonstrates that a real-time automated pain detection system (FPAS) based on facial expression analysis, incorporating parallel processing of action units and emotion recognition with individual baseline calibration, can achieve strong correlation and acceptable agreement with patient self-reported VAS assessments in postoperative PACU patients. These results support the potential utility of automated facial analysis as a supplementary tool for pain monitoring, particularly in patients who cannot verbally communicate.

The system should be considered a promising research tool and screening adjunct rather than a fully validated clinical instrument ready for routine implementation. Future work should focus on improving severe pain detection, validating the system in diverse clinical populations and settings, and integrating the system into nursing workflows with appropriate clinical decision support.

## Data Availability

The dataset presented in the study is available on request from the corresponding author during submission or after publication.
